# Newspapers and Periodicals

**Published:** 1856-12

**Authors:** 


### NEWSPAPERS AND PERIODICALS.

Whose mind does not run back to the sunnier days of childhood at the repetition of the dear familiar lines:

> "In works of labor or of skill,
> I would be busy too;
> For Satan finds some mischief still,
> For idle hands to do."

And how much better, too, is the good old Presbyterian custom of causing children to commit to memory such plain and wholesome truths, than of lumbering up their brains with the doggerel rhymes of *Old Mother Goose*, and such as

> "There was an old woman, she lived in a spoon,
> And all she wanted was elbow room."

Then again:

> "O, Miss Mary, quite contrary,
> How doos your garden grow?"
> "Silver bells and muscle shells,
> And cucumbers all in a row."

Eminently suggestive are such lines of—nonsense.

But what connection is there between newspapers and idle children? There is no connection whatever, reader, and that is precisely the point we are trying to make. We wish to express it as a mature conviction of our own mind, that one of the best protections for our children against the temptations of city and village life, is the habitual reading of a well-conducted family newspaper or periodical. If you want a child to take an interest in a paper, let it be *his* paper, sent to his address. In a reasonable time he will get to look for its coming, and feel the want of it, if it does not arrive at the usual time. Soon it will be a kind of necessity, and rather than be without it he becomes willing to make sacrifices and self-denials for the sake of saving any stray dime or half-dime which may happen to come into his possession. Peanuts and gingerbread, monkey-shows and fire-crackers, are vetoed, and the increment of a quarter of a dollar to a half, and so on, to the subscription price, is watched with an interest and a pleasure which few would imagine, and lo! the germs of an economy and a self-denial are planted before we are aware of it, which will grow to health, and wealth, and position.

The moment any child has learned to save, that moment such a child is rendered safe for life; safe from the penitentiary, safe from the poor-house, safe from her whose chambers go down to death. Not only so; during this time, the lessons learned from week to week, inculcated in short articles of precept and of fact—lessons in history, in finance, in morals, are indelibly impressed on the mind, and help to build up a character, which, with others like it, is to hold up the society of the next generation. Such being the case, no small responsibilities to our common country rest on the editorial profession; and glad are we, that to be well informed, to be educated, is the first, the essential requisite, in an editor; without it, he cannot keep his head above water. In law, physic, and divinity, an ignoramus may sail with the wind, may float on the tide of family, fortune, or party; but the only life-boat of a living editor is actual intelligence—no sham can live an hour.

Be assured, reader, that the price of a periodical for each child in your family, who has entered the tenth year, is an investment which will yield a dividend of a million per cent. The very idea of "*taking a paper*" elevates a child, increases his self-respect, and that feeling of self-importance which is the germ of manly and womanly dignity.

Truth, knowledge, has an infectious influence about it. The possession of one item of intelligence leads to the desire of knowing a kindred truth; this stimulates to investigation, when proper facilities, and encouragements, and aids are afforded; and all at once, we find the child an investigator, with an interest which insures its remembrance; and here we have a student *in embryo*—a self-taught scholar, the very kind of persons who, the world over, make the men of note of their time.

We have been a very long time in coming to the point of inviting parents, who take this journal, to look over the list of exchanges, on the last page of the cover, and make a good beginning for eighteen hundred and fifty-seven, by selecting some publication for each member of your family—that is, one for each child, one for your wife, and one, at least, for yourself—then, a good religious paper for the whole household. Perhaps you would do well to let each one choose for himself, subject to your superior judgment; then, count up the advance subscription price of all, and send it right away, to commence from and after the first day of January, eighteen hundred and fifty-seven, and their renewal every year will be among the pleasant events of "*Christmas Times*," which will not easily be forgotten; then, the editors will be sure of their money, and you sure of your papers; thus enabling them to labor cheerily, while you and all yours are reaping weekly gratification and instruction.

Two suggestions we give you as a matter of opinion only :—

1st. Give preference to the publication of the kind you want, which is nearest to you. "Local papers," as they are called, are of more importance than is generally supposed, when they are industriously edited. They keep you acquainted with the history of things around you, of the growth of your village or county, their improvements, changes, and the like. One of the most deeply interesting volumes in any man's library is a regular file of his village newspaper of twenty, of forty years agone. What reminiscences—how glad are some! others, how sweetly sad are they! And what a photographic panorama is given of the whole past!

Here we step aside to say to the editors of local papers, it will add to your permanent and best interests, to spend less time in scissoring city papers, and more in being "around" your own diocese, talking with the old people, hunting out early incidents of local interest, making yourself at the same time familiar with what Sam and Young America are about. In this way you, almost any one of you, will add a large percentage to your subscription list every year. Try it for a single year, gentlemen, and neither you nor your neighbors will ever repent of it.

Another suggestion: do not allow one member of the family to read any other paper than his own. This will have several very beneficial effects, not thought of by the superficial.

1st. It will prevent the very bad habit of borrowing a newspaper. Why! I would rather lend my umbrella, and even my dinner—*sometimes!*

2d. It is not profitable to read a variety of newspapers, no more than it is to hear a variety of preachers. We never saw a good Christian made by the latter practice, nor a head well filled by the former.

One paper well read, is more profitable than to have the skimming of a dozen; which last gives a kind of general diffusive knowledge, which is the farthest possible from being practical, and practical knowledge is the great want of the age. It is the knowledge of minutiæ, which is remunerative. While we would confine each one to the actual reading of his own paper, we would allow him to tell its news to the others. Does not any one know what pleasure it affords to tell to another what is supposed to be new? The desire to tell would induce greater care in impressing upon the mind the particulars of what was intended to be communicated, and this would cultivate a habit of minuteness, and accuracy of narration, which gives to conversation its instructiveness and its charm.

Then, again, a love of conversation is engendered, as to the useful and the true; a facility in expressing ideas grow up, which is invaluable; and we are never pained with the blunder of the pretender, "I have the idea, but can't express it satisfactorily." The fact is, an idea which can't find a medium of expression in words, is as empty as the head which holds it.

Suppose, then, a family should be of a size which would allow of the taking of some publication every day; we can scarcely imagine a more agreeable occupation for a winter's evening, than all gathering around the fire, and the father or mother, or eldest child taking the lead, to draw out the recipient of that day's paper, with the various side-issues connected with it.

Parents of New York and Philadelphia, and other large cities, it is a fault which has broken many hearts among you, that you *failed to make home inviting to your children!* and your sons sought amusement in the streets, or worse places; and your daughters in parties, with their frivolity, and heated rooms, and late suppers, and thin shoes, and gossamer dress—and the son, where is he? the habitué of the club-house or billiard room, or lower down still: and your daughter—let the combination of the lily and the hectic tell.

